# Genetic Analysis Reveals a Significant Contribution of *CES1* to Prostate Cancer Progression in Taiwanese Men

**DOI:** 10.3390/cancers12051346

**Published:** 2020-05-25

**Authors:** Chien-Chih Ke, Lih-Chyang Chen, Chia-Cheng Yu, Wei-Chung Cheng, Chao-Yuan Huang, Victor C. Lin, Te-Ling Lu, Shu-Pin Huang, Bo-Ying Bao

**Affiliations:** 1 Department of Medical Imaging and Radiological Sciences, Kaohsiung Medical University, Kaohsiung 807, Taiwan; ccke@kmu.edu.tw; 2 Department of Medical Research, Kaohsiung Medical University Hospital, Kaohsiung 807, Taiwan; 3 Department of Medicine, Mackay Medical College, New Taipei City 252, Taiwan; lihchyang@mmc.edu.tw; 4 Division of Urology, Department of Surgery, Kaohsiung Veterans General Hospital, Kaohsiung 813, Taiwan; ccyu@vghks.gov.tw; 5 Department of Urology, School of Medicine, National Yang-Ming University, Taipei 112, Taiwan; 6 Department of Pharmacy, College of Pharmacy and Health Care, Tajen University, Pingtung 907, Taiwan; 7 Graduate Institute of Biomedical Sciences, China Medical University, Taichung 404, Taiwan; wccheng@mail.cmu.edu.tw; 8 Research Center for Tumor Medical Science, China Medical University, Taichung 404, Taiwan; 9 Drug Development Center, China Medical University, Taichung 404, Taiwan; 10 Department of Urology, National Taiwan University Hospital, College of Medicine, National Taiwan University, Taipei 100, Taiwan; cyhuang0909@ntu.edu.tw; 11 Department of Urology, E-Da Hospital, Kaohsiung 824, Taiwan; victorlin0098@yahoo.com.tw; 12 School of Medicine for International Students, I-Shou University, Kaohsiung 840, Taiwan; 13 Department of Pharmacy, China Medical University, Taichung 404, Taiwan; lutl@mail.cmu.edu.tw; 14 Department of Urology, Kaohsiung Medical University Hospital, Kaohsiung 807, Taiwan; 15 Graduate Institute of Medicine, College of Medicine, Kaohsiung Medical University, Kaohsiung 807, Taiwan; 16 Department of Urology, Faculty of Medicine, College of Medicine, Kaohsiung Medical University, Kaohsiung 807, Taiwan; 17 Center for Cancer Research, Kaohsiung Medical University, Kaohsiung 807, Taiwan; 18 Sex Hormone Research Center, China Medical University Hospital, Taichung 404, Taiwan; 19 Department of Nursing, Asia University, Taichung 413, Taiwan

**Keywords:** carboxylesterase, prostate cancer, progression, prognosis, biomarker

## Abstract

The genes that influence prostate cancer progression remain largely unknown. Since the carboxylesterase gene family plays a crucial role in xenobiotic metabolism and lipid/cholesterol homeostasis, we hypothesize that genetic variants in carboxylesterase genes may influence clinical outcomes for prostate cancer patients. A total of 478 (36 genotyped and 442 imputed) single nucleotide polymorphisms (SNPs) in five genes of the carboxylesterase family were assessed in terms of their associations with biochemical recurrence (BCR)-free survival in 643 Taiwanese patients with prostate cancer who underwent radical prostatectomy. The strongest association signal was shown in *CES1* (*P* = 9.64 × 10^−4^ for genotyped SNP rs8192935 and *P* = 8.96 × 10^−5^ for imputed SNP rs8192950). After multiple test correction and adjustment for clinical covariates, *CES1* rs8192935 (*P* = 9.67 × 10^−4^) and rs8192950 (*P* = 9.34 × 10^−5^) remained significant. These SNPs were correlated with *CES1* expression levels, which in turn were associated with prostate cancer aggressiveness. Furthermore, our meta-analysis, including eight studies, indicated that a high *CES1* expression predicted better outcomes among prostate cancer patients (hazard ratio 0.82, 95% confidence interval 0.70–0.97, *P* = 0.02). In conclusion, our findings suggest that *CES1* rs8192935 and rs8192950 are associated with BCR and that *CES1* plays a tumor suppressive role in prostate cancer.

## 1. Introduction

Prostate cancer is the most commonly diagnosed cancer and the second most common cause of death among men, with an estimated 191,930 new cases and 33,330 deaths expected worldwide in 2020 [1]. Standardized clinical management approaches, such as radical prostatectomy (RP), radiotherapy, and androgen deprivation therapy, have led to improved outcomes in patients with prostate cancer. However, prognosis remains heterogeneous, suggesting that genetic factors may contribute to treatment response. Genome-wide association studies (GWASs) have successfully identified more than 100 prostate cancer susceptibility loci to date [2,3]. Further functional studies indicate that these risk loci are often located near genes, and regulate genes involved in carcinogenesis, including cell metabolism (*JAZF1* and *HNF1B*) and DNA repair or cell cycle machinery (*MYC*, *TERT*, *ATM*, and *CDKN1B*) [4]. A pathway-based analysis using known prostate cancer susceptibility loci highlights the antigen presentation pathway and gene network of lipid metabolism, molecular transport, and small molecule biochemistry, which may contribute to prostate cancer development [5]. However, scanning for an association between genetic variants and the prognosis of prostate cancer is still difficult due to the need for a large sample size and long-term follow-up [6,7]. Despite a reasonably large cohort of 24,023 prostate cancer patients with 3513 disease-specific deaths, no evidence of association was observed between genetic variants and prostate cancer survival [7]. GWASs always focus on the most significant genetic variants, which may miss the loci that confer true effects but do not rank at the top. The biological hypothesis-driven approach allows for targeted evaluation and improves the power to detect significant associations. Several functional variants have been reported to be associated with prostate cancer survival by using this approach [8,9].

The carboxylesterase (CES) gene family encodes major liver enzymes, which are responsible for the hydrolysis of various endogenous substrates, including esters, thioesters, amides, carbamates, and xenobiotics, including toxins and drugs [10]. Five human CESs have been identified, and these enzymes share 39–46% of amino acid sequence identity [11]. Although CESs are expressed in most metabolic organs, indicating their protective roles against xenobiotics, they still exhibit different tissue distribution and substrate specificity. CES1 is mainly expressed in the liver and prefers to hydrolyze substrates containing a bulky acyl group and a small alcohol group, whereas CES2 is abundantly expressed in the small intestine and colon and prefers to metabolize esters with a small acyl group and a relatively large alcohol group [12,13]. CESs also appear to participate in the metabolism of fatty acids and cholesterol esters and play a role in the blood–brain barrier system [14], suggesting that the enzymes they encode for serve pivotal physiological functions. Interestingly, *CES* gene expression has been reported to be downregulated in certain cancer types as the diseases progress [15,16]. The expression of *CES* genes has also been shown to correlate with chemosensitivity in colorectal cancer [17,18]. A genetic analysis indicated a significant association between the single nucleotide polymorphism (SNP) rs11075646 in the 5' UTR of *CES2* and the response rate and time to progression in patients with cancer treated with capecitabine [19]. Therefore, we hypothesized that *CES* gene polymorphisms may also contribute to the differences in prostate cancer outcomes.

To date, no study has investigated whether CESs could mediate prostate cancer progression. In the present study, we analyzed SNP genotyping data and imputed unobserved SNPs in *CES* genes to comprehensively assess their impact on disease recurrence in prostate cancer patients who received RP.

## 2. Materials and Methods

### 2.1. Patient Recruitment and Data Collection

This study included 643 Taiwanese patients who underwent RP for localized prostate cancer at three medical centers in Taiwan: Kaohsiung Medical University Hospital, Kaohsiung Veterans General Hospital, and National Taiwan University Hospital, as described previously [20]. The clinicopathological data were obtained from the patients’ medical records. Biochemical recurrence (BCR) was defined as two consecutive prostate-specific antigen (PSA) elevation events of 0.2 ng/mL or more [21,22,23,24]. The protocol was approved by the institutional review board of Kaohsiung Medical University Hospital (KMUHIRB-2013132), and each participant provided written informed consent, in accordance with the ethical guidelines.

### 2.2. SNP Selection and Genotyping

We selected 36 haplotype tagging SNPs within the five *CES* genes and their 10 kb flanking regions with a threshold of a minor allele frequency (MAF) of >0.03, based on the 1000 Genomes data for Han Chinese in Beijing, China and Southern Han Chinese [25]. Genomic DNA was extracted from peripheral blood, and genotyping was conducted using Affymetrix Axiom Genotyping Arrays at the National Centre for Genome Medicine, Taiwan, as described previously [26]. The prediction of the untyped SNPs was performed using Minimac4 with 1000 Genomes Project Phase 3 East Asian reference panels [27,28]. SNPs were filtered by a MAF >0.03 and a Hardy–Weinberg equilibrium >0.001, resulting in 36 SNPs being genotyped and an additional 442 SNPs being imputed.

### 2.3. Bioinformatics Analysis

The functional prediction for the identified SNPs was performed with HaploReg v4.1 (https://pubs.broadinstitute.org/mammals/haploreg/haploreg.php) [29]. The expression quantitative trait loci (eQTL) analysis was determined using data from the Genotype–Tissue Expression (GTEx) project [30]. The associations between gene expression levels and prostate cancer survival were assessed using multiple data sources: GSE10645 [31], GSE116918 [32], GSE16560 [33], GSE21032 [34], GSE54460 [35], GSE70768 [36], GSE70769 [36], and The Cancer Genome Atlas (TCGA) [37] projects.

### 2.4. Statistical Analysis

Analyses were performed using Statistical Package for the Social Sciences software version 19.0.0 (IBM, Armonk, NY, USA). A two-sided *P* < 0.05 indicated statistical significance; *q* values were calculated for multiple test correction to report the false discovery rate [38].

## 3. Results

This analysis included 643 patients who underwent RP for localized prostate cancer. Their clinical characteristics were presented in Table 1. Two hundred and twenty-eight (35.5%) patients experienced BCR with a median follow-up time of 51 months. Univariate Cox regression indicated that PSA, Gleason score, stage, and surgical margin were significantly associated with BCR (*P* < 0.001).

**Figure 1:**
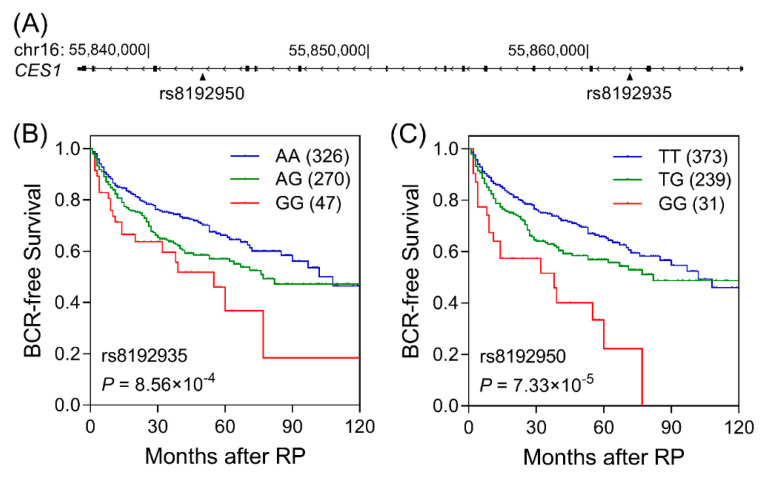
Association of *CES1* rs8192935 and rs8192950 with biochemical recurrence (BCR)-free survival. (**A**) Schematic genomic structure of *CES1* and the locations of rs8192935 and rs8192950. Kaplan–Meier curves of BCR-free survival for rs8192935 (**B**) and rs8192950 (**C**) genotypes. Values in brackets denote the number of patients. RP, radical prostatectomy.

We performed a single-locus Cox regression analysis to assess the associations of 36 genotyped SNPs in the five *CES* genes with BCR. Three SNPs were found to be associated with BCR (*P* < 0.05, Table S1), of which *CES1* rs8192935 remained noteworthy after multiple test correction (*q* = 0.036). We sought to identify the SNPs better correlated with BCR through imputation, referencing the 1000 Genomes Project (East Asian population). Of the additional 442 SNPs that passed imputation quality control, six SNPs showed superior associations with BCR compared to rs8192935. The strongest signal was shown by rs8192950, which is in linkage disequilibrium with rs8192935 (*r*^2^ = 0.753). The risk of BCR was significantly increased with the number of *CES1* rs8192935 G and rs8192950 G alleles (*P* = 9.64 × 10^−4^ and 8.96 × 10^−5^, respectively, Table 2 and Figure 1). Additionally, these two SNPs remained independently and significantly associated with BCR after adjustment for age, PSA, Gleason score, cancer stage, and surgical margin (hazard ratio (HR) 1.43, 95% confidence interval (CI) 1.16–1.76, *P* = 9.67 × 10^−4^ for rs8192935, and HR 1.50, 95% CI 1.24–1.90, *P* = 9.34 × 10^−5^ for rs8192950, Table 2).

**Figure 2:**
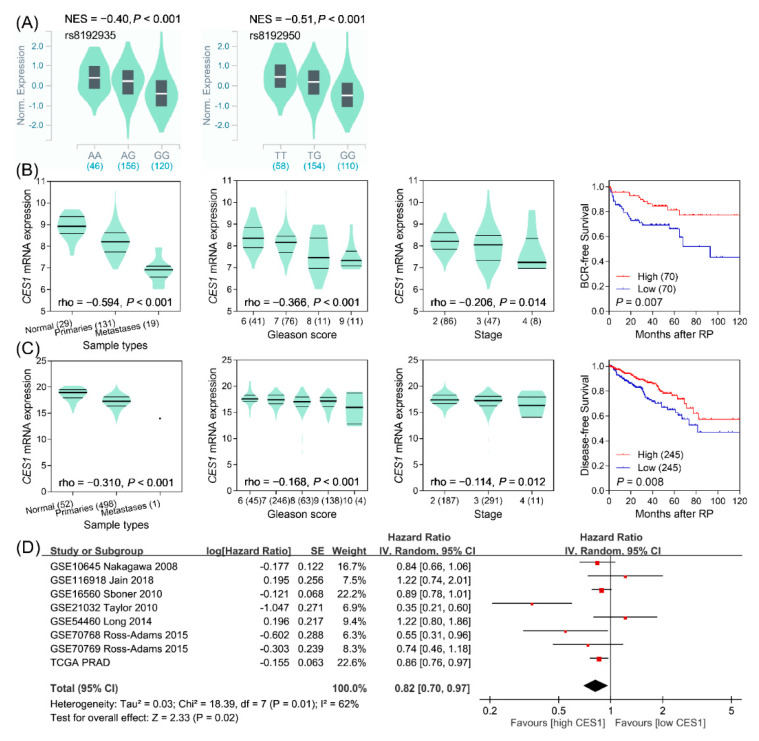
Association of *CES1* polymorphisms with prostate cancer progression. (**A**) The correlation of rs8192935 (left) and rs8192950 (right) genotypes with *CES1* mRNA expression levels in testis tissues from the Genotype–Tissue Expression database. NES, normalized effect size. Lower expression of *CES1* correlates with prostate cancer, a higher Gleason score and stage, and poorer patient prognosis in the Taylor cohort (**B**), as well as in The Cancer Genome Atlas (TCGA) cohort (**C**). BCR, biochemical recurrence. RP, radical prostatectomy. rho, Spearman's rank correlation coefficient. (**D**) Meta-analysis of eight studies evaluating the hazard ratio of high compared with low levels of *CES1* mRNA expression for prostate cancer prognosis. SE, standard error. IV, inverse variance.

To identify the possible effects of these SNPs, functional annotations were extracted from the HaploReg v4.1. Both rs8192935 and rs8192950 have effects on enhancer histone marks and motif alterations and are eQTL SNPs for *CES1* (Table S2). The rs8192935 G and rs8192950 G alleles were associated with lower expression levels of *CES1* in 322 testis tissues from the GTEx Project (Figure 2A). However, the SNPs are not correlated with expression in prostate tissues, possibly due to the small sample size (Figure S1). These results indicated that lower *CES1* expression would correlate with a poor prognosis in prostate cancer. According to two prostate cancer studies from Taylor and TCGA, lower expression levels of *CES1* were associated with prostate cancer, a higher Gleason score, a more advanced stage, and worse survival in patients (Figure 2B,C). A meta-analysis of eight cohorts of 2064 prostate cancer patients was performed to further evaluate the prognostic significance of *CES1*. The results showed that higher *CES1* expression was significantly related to better prostate cancer prognosis under a random effects model (HR 0.82, 95% CI 0.70–0.97, *P* = 0.02, Figure 2D).

## 4. Discussion

In the present study, we genotyped haplotype-tagging SNPs in *CES* family genes, following imputation, to fine-map additional SNPs that may be relevant and comprehensively analyze their association with prostate cancer progression. We found that *CES1* rs8192935 and rs8192950 might be a prognostic factor for BCR-free survival in patients with prostate cancer. Furthermore, functional studies revealed that these SNPs are in eQTL affecting the expression of *CES1* and are subsequently correlated with tumor aggressiveness and prostate cancer prognosis.

CES enzymes are primarily localized within the endoplasmic reticulum in many tissues and play key roles in both endobiotic metabolism and the activation/detoxification of xenobiotics [10]. CES1, also known as serine esterase 1 or monocyte esterase and cholesterol ester hydrolase, is abundantly produced in the epithelia of metabolic organs including the liver, lungs, and bladder, indicating its protective role against xenobiotics [39]. Interestingly, the inhibition of CES1 in monocytes was shown to diminish their ability to lyse tumor cells [40]. The increased frequency of deficient CES1 enzyme activity has also been reported in non-Hodgkin lymphoma and B-cell chronic lymphocytic leukemia [41,42]. These findings suggest a possible tumor-cell-killing or surveillance function of CES1. Furthermore, *CES1* is also a transcriptional target gene of pregnane X receptor (PXR) [43], a key sensor of the body’s defense mechanism against xenobiotics. The activation of PXR was found to markedly lower the concentration of circulating androgens, suppress prostate regeneration, and inhibit the growth of human prostate cancer cells [44]. The role of PXR in the homeostasis of androgens may provide clues to the mechanism underlying the observed association between CES1 and prostate cancer progression. In the present study, we found that carriers of the *CES1* rs8192935 and rs8192950 G variants had a worse BCR-free survival. Since CES1 is the major enzyme responsible for the hydrolysis of many clinical drugs, *CES1* rs8192935 and rs8192950 have been recognized as important pharmacogenetic regulators of treatment outcomes [45,46]. According to the annotation of HaploReg, these two variants may be functional, as they are located at the enhancer region and eQTL of *CES1*, and are likely to disrupt transcription factor binding motifs in various cells. Consistently, we found that rs8192935 and rs8192950 G alleles were associated with a decrease in the mRNA expression levels of *CES1*, and lower *CES1* expression showed a poorer prognosis for prostate cancer patients. However, these SNPs did not affect *CES1* expression in prostate tissue, probably because of the limited prostate samples in the GTEx database. Therefore, further experimental characterization is required to elucidate the function of these SNPs/CES1 in prostate cancer.

This study has several inherent limitations. All the participants in our cohort are Taiwanese, and the findings may not be generalizable to other ethnic groups. Although multiple test correction was performed, the current results still have to be interpreted with caution. In addition, this is a retrospective study with a moderate sample size, and large studies with prospective designs are needed to validate our findings. Finally, no direct biological experiments were conducted to investigate the exact mechanism of action of *CES1* rs8192935 and rs8192950 on prostate cancer progression, which should be explored in the future.

## 5. Conclusions

Our results suggest that rs8192935 and rs8192950 may reduce the expression of *CES1*, resulting in a poor prognosis, and could be potential biomarkers of clinical outcome in prostate cancer patients. However, validation in a larger population and further functional studies are needed to identify the tumor suppressive role of *CES1* underlying prostate cancer progression.

## Figures and Tables

**Table 1:** Clinicopathologic characteristics of the study population.

Characteristics	*n*	BCR, *n* (%)	HR (95% CI)	*P*
Age at diagnosis, years				
Median (IQR)	66.0 (62.0–70.0)			
≤66	331	115 (34.7)	1.00	
>66	312	113 (36.2)	1.08 (0.83–1.40)	0.552
PSA at diagnosis, ng/mL^a^				
Median (IQR)	10.9 (7.02–18.41)			
≤10	292	74 (25.3)	1.00	
>10	330	145 (43.9)	2.23 (1.68–2.95)	<0.001
Gleason score				
2–7	531	164 (30.9)	1.00	
8–10	112	64 (57.1)	2.81 (2.10–3.76)	<0.001
Stage ^a^				
T1/T2	363	88 (24.2)	1.00	
T3/T4/N1	275	136 (49.5)	2.79 (2.13–3.65)	<0.001
Surgical margin				
Negative	459	139 (30.3)	1.00	
Positive	184	89 (48.4)	2.02 (1.55–2.65)	<0.001
Total	643	228 (35.5)		

Abbreviations: BCR, biochemical recurrence; HR, hazard ratio; CI, confidence interval; IQR, interquartile range; PSA, prostate-specific antigen. ^a^ Some subtotals do not sum to 643 due to missing data.

**Table 2:** SNPs associated with BCR in prostate cancer patients receiving RP.

Gene SNP	Position	Genotype	Frequency	BCR	HR (95% CI)	*P*	HR (95% CI) ^a^	*P* ^a^
*CES1* rs8192935 ^b^	55861794	AA/AG/GG	326/270/47	100/106/22	1.41 (1.15–1.72)	9.64 × 10^−4^	1.43 (1.16–1.76)	9.67 × 10^−4^
*CES1* rs8192950	55842404	TT/TG/GG	373/239/31	114/95/19	1.53 (1.24–1.89)	8.96 × 10^−5^	1.50 (1.24–1.90)	9.34 × 10^−5^

Abbreviations: SNP, single nucleotide polymorphism; BCR, biochemical recurrence; RP, radical prostatectomy; HR, hazard ratio; CI, confidence interval. ^a^ Adjustment for age, PSA at diagnosis, Gleason score, stage, and surgical margin. ^b^ Genotyped SNP.

## Supplementary Materials

Supplementary materials can be found at https://www.mdpi.com/2072-6694/12/5/1346/s1. Table S1: Genotyped SNPs and the *P* values of their association with BCR after RP. Table S2: Regulatory annotation of *CES1* rs8192935 and rs8192950. Figure S1: The correlation of rs8192935 and rs8192950 genotypes with *CES1* mRNA expression levels in prostate tissues from the GTEx database.

## References

[1] Siegel R.L., Miller K.D., Jemal A. (2020). Cancer statistics, 2020. Ca Cancer J. Clin..

[2] Amundadottir L.T., Sulem P., Gudmundsson J., Helgason A., Baker A., Agnarsson B., Sigurdsson A., Benediktsdottir K.R., Cazier J.-B., Sainz J. (2006). A common variant associated with prostate cancer in European and African populations. Nat. Genet..

[3] Yeager M., Orr N., Hayes R.B., Jacobs K., Kraft P., Wacholder S., Minichiello M.J., Fearnhead P., Yu K., Chatterjee N. (2007). Genome-wide association study of prostate cancer identifies a second risk locus at 8q24. Nat. Genet..

[4] Schumacher F.R., Al Olama A.A., Berndt S.I., Benlloch S., Ahmed M., Saunders E., Dadaev T., Leongamornlert D., Anokian E., Cieza-Borrella C. (2018). Association analyses of more than 140,000 men identify 63 new prostate cancer susceptibility loci. Nat. Genet..

[5] Farashi S., Kryza T., Clements J., Batra J. (2018). Post-GWAS in prostate cancer: From genetic association to biological contribution. Nat. Rev. Cancer.

[6] Li W., Middha M., Bicak M., Sjoberg D., Vertosick E., Dahlin A., Häggström C., Hallmans G., Rönn A.-C., Stattin P. (2018). Genome-wide Scan Identifies Role for AOX1 in Prostate Cancer Survival. Eur. Urol..

[7] Szulkin R., Karlsson R., Whitington T., Aly M., Gronberg H., Eeles R., Easton U.F., Kote-Jarai Z., Al Olama A.A., Benlloch S. (2015). Genome-wide association study of prostate cancer-specific survival. Cancer Epidemiol. Biomark. Prev..

[8] Huang C.-N., Huang S.-P., Pao J.-B., Chang T.-Y., Lan Y.-H., Lu T.-L., Lee H.-Z., Juang S.-H., Wu P.-P., Pu Y.-S. (2012). Genetic polymorphisms in androgen receptor-binding sites predict survival in prostate cancer patients receiving androgen-deprivation therapy. Ann. Oncol..

[9] Lindström S., Adami H.-O., Bälter K.A., Xu J., Zheng S.L., Stattin P., Grönberg H., Wiklund F. (2007). Inherited Variation in Hormone-Regulating Genes and Prostate Cancer Survival. Clin. Cancer Res..

[10] Ross M., Crow J.A. (2007). Human carboxylesterases and their role in xenobiotic and endobiotic metabolism. J. Biochem. Mol. Toxicol..

[11] Holmes R.S., Wright M., Laulederkind S.J.F., Cox L.A., Hosokawa M., Imai T., Ishibashi S., Lehner R., Miyazaki M., Perkins E.J. (2010). Recommended nomenclature for five mammalian carboxylesterase gene families: Human, mouse, and rat genes and proteins. Mamm. Genome.

[12] Hosokawa M. (2008). Structure and Catalytic Properties of Carboxylesterase Isozymes Involved in Metabolic Activation of Prodrugs. Molecules.

[13] Sanghani S.P., Sanghani P.C., Schiel M.A., Bosron W. (2009). Human carboxylesterases: An update on CES1, CES2 and CES3. Protein Pept. Lett..

[14] Ose A., Kusuhara H., Yamatsugu K., Kanai M., Shibasaki M., Fujita T., Yamamoto A., Sugiyama Y. (2007). P-glycoprotein Restricts the Penetration of Oseltamivir Across the Blood-Brain Barrier. Drug Metab. Dispos..

[15] Cai L., Tang X., Guo L., An Y., Wang Y., Zheng J. (2009). Decreased serum levels of carboxylesterase-2 in patients with ovarian cancer. Tumori J..

[16] Tang X., Wu H., Wu Z., Wang G., Wang Z., Zhu D. (2008). Carboxylesterase 2 is Downregulated in Colorectal Cancer Following Progression of the Disease. Cancer Investig..

[17] Cecchin E., Corona G., Masier S., Biason P., Cattarossi G., Frustaci S., Buonadonna A., Colussi A., Toffoli G. (2005). Carboxylesterase Isoform 2 mRNA Expression in Peripheral Blood Mononuclear Cells Is a Predictive Marker of the Irinotecan to SN38 Activation Step in Colorectal Cancer Patients. Clin. Cancer Res..

[18] Chiorean E.G., Sanghani S., Schiel M.A., Yu M., Burns M., Tong Y., Hinkle D.T., Coleman N., Robb B., Leblanc J. (2012). Phase II and gene expression analysis trial of neoadjuvant capecitabine plus irinotecan followed by capecitabine-based chemoradiotherapy for locally advanced rectal cancer: Hoosier Oncology Group GI03-53. Cancer Chemother. Pharmacol..

[19] Ribelles N., Lopez-Siles J., Sanchez A., Gonzalez E., Sánchez M.J., Carabantes F., Sanchez-Rovira P., Marquez A., Duenas R., Sevilla I. (2008). A carboxylesterase 2 gene polymorphism as predictor of capecitabine on response and time to progression. Curr. Drug Metab..

[20] Huang S.-P., Huang L.-C., Ting W.-C., Chen L.M., Chang T.-Y., Lu T.-L., Lan Y.-H., Liu C.-C., Yang W.-H., Lee H.-Z. (2009). Prognostic Significance of Prostate Cancer Susceptibility Variants on Prostate-Specific Antigen Recurrence after Radical Prostatectomy. Cancer Epidemiol. Biomark. Prev..

[21] Freedland S.J., E Sutter M., Dorey F., Aronson W.J. (2003). Defining the ideal cutpoint for determining PSA recurrence after radical prostatectomy. Urology.

[22] Huang C.-Y., Huang S.-P., Lin V.C., Yu C.-C., Chang T.-Y., Juang S.-H., Bao B.-Y. (2015). Genetic variants in the Hippo pathway predict biochemical recurrence after radical prostatectomy for localized prostate cancer. Sci. Rep..

[23] Huang E.Y., Chang Y.-J., Huang S.-P., Lin V.C., Yu C.-C., Huang C.-Y., Yin H.-L., Chang T.-Y., Lu T.-L., Bao B. (2018). A common regulatory variant in SLC 35B4 influences the recurrence and survival of prostate cancer. J. Cell. Mol. Med..

[24] Huang S.-P., Lévesque E., Guillemette C., Yu C.-C., Huang C.-Y., Lin V.C., Chung I.-C., Chen L.-C., Laverdière I., Lacombe L. (2014). Genetic variants in microRNAs and microRNA target sites predict biochemical recurrence after radical prostatectomy in localized prostate cancer. Int. J. Cancer.

[25] Abecasis G.R., Auton A., Brooks L.D., de Pristo M.A., Durbin R.M., Handsaker R.E., Kang H.M., Marth G.T., McVean G.A., 1000 Genomes Project Consotium (2012). An integrated map of genetic variation from 1092 human genomes. Nature.

[26] Huang C.-N., Huang S.-P., Pao J.-B., Hour T.-C., Chang T.-Y., Lan Y.-H., Lu T.-L., Lee H.-Z., Juang S.-H., Wu P.-P. (2011). Genetic polymorphisms in oestrogen receptor-binding sites affect clinical outcomes in patients with prostate cancer receiving androgen-deprivation therapy. J. Intern. Med..

[27] Das S., Forer L., Schönherr S., Sidore C., Locke A.E., Kwong A., I Vrieze S., Chew E.Y., Levy S., McGue M. (2016). Next-generation genotype imputation service and methods. Nat. Genet..

[28] Li Y., Willer C.J., Ding J., Scheet P., Abecasis G. (2010). MaCH: Using sequence and genotype data to estimate haplotypes and unobserved genotypes. Genet. Epidemiol..

[29] Ward L.D., Kellis M. (2015). HaploReg v4: Systematic mining of putative causal variants, cell types, regulators and target genes for human complex traits and disease. Nucleic Acid. Res..

[30] Consortium G.T. (2013). The Genotype-Tissue Expression (GTEx) project. Nat. Genet..

[31] Nakagawa T., Kollmeyer T.M., Morlan B.W., Anderson S.K., Bergstralh E.J., Davis B.J., Asmann Y.W., Klee G.G., Ballman K., Jenkins R.B. (2008). A Tissue Biomarker Panel Predicting Systemic Progression after PSA Recurrence Post-Definitive Prostate Cancer Therapy. PLoS ONE.

[32] Jain S., Lyons C., Walker S., McQuaid S., Hynes S., Mitchell D., Pang B., Logan G., McCavigan A., O’Rourke D. (2018). Validation of a Metastatic Assay using biopsies to improve risk stratification in patients with prostate cancer treated with radical radiation therapy. Ann. Oncol..

[33] Sboner A., Demichelis F., Calza S., Pawitan Y., Setlur S.R., Hoshida Y., Perner S., Adami H.-O., Fall K., Mucci L. (2010). Molecular sampling of prostate cancer: A dilemma for predicting disease progression. BMC Med. Genom..

[34] Taylor B.S., Schultz N., Hieronymus H., Gopalan A., Xiao Y., Carver B.S., Arora V.K., Kaushik P., Cerami E., Reva B. (2010). Integrative Genomic Profiling of Human Prostate Cancer. Cancer Cell.

[35] Long Q., Xu J., Osunkoya A.O., Sannigrahi S., Johnson B.A., Zhou W., Gillespie T., Park J.Y., Nam R.K., Sugar L. (2014). Global transcriptome analysis of formalin-fixed prostate cancer specimens identifies biomarkers of disease recurrence. Cancer Res..

[36] Ross-Adams H., Lamb A.D., Dunning M., Halim S., Lindberg J., Massie C.E., Egevad L., Russell R., Ramos-Montoya A., Vowler S. (2015). Integration of copy number and transcriptomics provides risk stratification in prostate cancer: A discovery and validation cohort study. EBioMedicine.

[37] Cancer Genome Atlas Research Network (2008). Comprehensive genomic characterization defines human glioblastoma genes and core pathways. Nature.

[38] Storey J., Tibshirani R. (2003). Statistical significance for genomewide studies. Proc. Nat. Acad. Sci. USA.

[39] Taketani M., Shii M., Ohura K., Ninomiya S., Imai T. (2007). Carboxylesterase in the liver and small intestine of experimental animals and human. Life Sci..

[40] McCormick J.A., Markey G.M., Morris T.C.M., Auld P.W., Alexander H.D. (1991). Lactoferrin inducible monocyte cytotoxicity defective in esterase deficient monocytes. Br. J. Haematol..

[41] Burgaleta C., Villalba S., González N. (1999). Defective activity of monocytes from patients with non-Hodgkin lymphoma. The modulatory effect of granulocyte-macrophage-colony stimulating factor. Cancer.

[42] Markey G.M., A McCormick J., Morris T.C., Alexander H.D., Nolan L., Morgan L.M., E Reynolds M., Edgar S., Bell A.L., McCaigue M.D. (1990). Monocyte esterase deficiency in malignant neoplasia. J. Clin. Pathol..

[43] Yang J., Yan B. (2006). Photochemotherapeutic Agent 8-Methoxypsoralen Induces Cytochrome P450 3A4 and Carboxylesterase HCE2: Evidence on an Involvement of the Pregnane X Receptor. Toxicol. Sci..

[44] Zhang B., Cheng Q., Ou Z., Lee J.H., Xu M., Kochhar U., Ren S., Huang M., Pflug B.R., Xie W. (2010). Pregnane X receptor as a therapeutic target to inhibit androgen activity. Endocrinology.

[45] di Matteo C., D’Andrea G., Vecchione G., Paoletti O., Cappucci F., Tiscia G.L., Buono M., Grandone E., Testa S., Margaglione M. (2016). Pharmacogenetics of dabigatran etexilate interindividual variability. Thromb. Res..

[46] Zhao Z., Li X., Sun S., Mei S., Ma N., Miao Z., Peng S. (2016). Impact of genetic polymorphisms related to clopidogrel or acetylsalicylic acid pharmacology on clinical outcome in Chinese patients with symptomatic extracranial or intracranial stenosis. Eur. J. Clin. Pharmacol..

