# Supplementary material for: Genetic Analysis Reveals a Significant Contribution of CES1 to Prostate Cancer Progression in Taiwanese Men
Source: Cancers (Basel). 2020 May 25;12(5):1346. doi: 10.3390/cancers12051346 (PMC7281132; doi:10.3390/cancers12051346)
Supplement: Supplementary file 1 [file cancers-12-01346-s001.pdf]

Table 1. Genotyped SNPs and the *P* values of their association with BCR after RP.

Gene	SNP ID	Chromosome	Position	HWE	MAF	Alleles	*P*	*q*
CES1	rs28709456	16	55842641	0.050	0.185	A > C	0.035	0.468
CES1	rs4513095	16	55853446	0.502	0.061	C > A	0.289	0.839
CES1	rs75990781	16	55859192	0.487	0.093	C > A	0.394	0.839
CES1	rs8192936	16	55860678	0.091	0.186	C > T	0.039	0.468
CES1	rs8192935	16	55861794	0.385	0.282	A > G	0.001	**0.036**
CES1	rs2307240	16	55862712	1.000	0.050	C > T	0.411	0.839
CES1	rs4783904	16	55865195	0.049	0.478	T > G	0.661	0.839
CES1	rs6499786	16	55869235	0.295	0.421	A > G	0.599	0.839
CES5A	rs1861318	16	55877700	0.034	0.266	G > A	0.623	0.839
CES5A	rs7184362	16	55879620	0.701	0.053	A > G	0.788	0.915
CES5A	rs11860488	16	55880534	0.331	0.238	G > C	0.340	0.839
CES5A	rs1968174	16	55882577	0.638	0.211	C > A	0.345	0.839
CES5A	rs4783914	16	55886662	1.000	0.116	A > G	0.841	0.929
CES5A	rs9937572	16	55890398	0.676	0.377	G > C	0.956	0.975
CES5A	rs147413705	16	55899404	0.618	0.041	G > A	0.340	0.839
CES5A	rs11076128	16	55899703	0.466	0.252	T > C	0.709	0.851
CES5A	rs7405407	16	55901189	1.000	0.148	T > C	0.152	0.839
CES5A	rs7499570	16	55902957	0.499	0.135	G > A	0.413	0.839
CES5A	rs114166221	16	55906042	0.595	0.037	G > T	0.649	0.839
CES5A	rs118152253	16	55906246	0.594	0.080	C > T	0.586	0.839
CES5A	rs12597310	16	55906358	0.610	0.189	C > G	0.271	0.839
CES5A	rs8056747	16	55906487	0.463	0.402	A > C	0.128	0.839
CES5A	rs28623213	16	55906782	0.750	0.066	C > G	0.183	0.839
CES5A	rs75723774	16	55908971	0.479	0.126	G > A	0.578	0.839
CES5A	rs28493887	16	55910488	0.072	0.140	A > G	0.676	0.839
CES5A	rs4784604	16	55922429	0.172	0.114	A > G	0.975	0.975
CES5A	rs11645591	16	55928523	0.028	0.217	T > C	0.608	0.839
CES5A	rs72810534	16	55944095	1.000	0.080	T > C	0.307	0.839
CES5A	rs12596847	16	55953246	0.052	0.079	G > A	0.562	0.839
CES5A	rs76052780	16	55954541	0.026	0.111	T > G	0.852	0.929
CES2	rs2241410	16	66972373	0.349	0.066	C > A	0.344	0.839
CES2	rs146988162	16	66983545	0.514	0.033	C > T	0.159	0.839
CES3	rs79945493	16	66988686	1.000	0.042	C > G	0.964	0.975
CES3	rs574288305	16	67014415	0.611	0.038	G > A	0.588	0.839
CES4A	rs78020902	16	67036161	0.143	0.140	C > T	0.585	0.839
CES4A	rs61744399	16	67037054	0.621	0.198	A > C	0.594	0.839

Abbreviations: SNP, single nucleotide polymorphism; BCR, biochemical recurrence; RP, radical prostatectomy; MAF, minor alleles frequency; HWE, Hardy-Weinberg equilibrium. $q < 0.05$ is in boldface.

Table 2. Regulatory annotation of *CES1* rs8192935 and rs8192950.

Chromosome	Position	SNP ID	LD (r^2)	Reference allele	Alternate allele	ASN frequency	Variant type	Enhancer histone marks	eQTL hits	Motifs changed
16	55861794	rs8192935	1	A	G	0.23	intronic	LIV	2 hits	Foxj2, PLZF, Pou5f1, p300
16	55842404	rs8192950	1	T	G	0.19	intronic	ESC, IPSC, LIV	3 hits	Crx, Evi-1, HNF1, Hoxa4, Hoxa5, Pax-4, Pou2f2

Abbreviations: LD, linkage disequilibrium; eQTL, Expression quantitative trait loci.

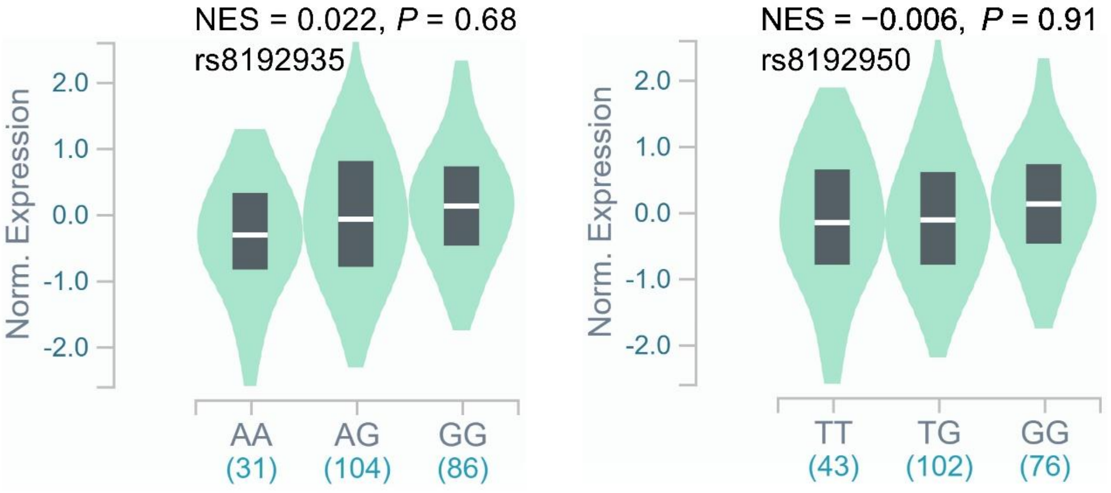

Figure 1. The correlation of rs8192935 and rs8192950 genotypes with *CES1* mRNA expression levels in prostate tissues from the GTEx database. NES, normalized effect size.
